# Does phone messaging improves tuberculosis treatment success? A systematic review and meta-analysis

**DOI:** 10.1186/s12879-020-4765-x

**Published:** 2020-01-14

**Authors:** Kassahun Dessie Gashu, Kassahun Alemu Gelaye, Zeleke Abebaw Mekonnen, Richard Lester, Binyam Tilahun

**Affiliations:** 1 Department of Health Informatics, Institute of Public Health, College of Medicine and Health Sciences, University of Gondar, Gondar, Ethiopia; 2 Department of Epidemiology and Biostatistics, Institute of Public Health, College of Medicine and Health Science, University of Gondar, Gondar, Ethiopia; 3 Research Pavilion, Rm 566, 828 W 10th, University of British Columbia, Vancouver, BC V5Z 1 M9 Canada

**Keywords:** Mobile phone, Treatment success, Text messaging, Tuberculosis

## Abstract

**Background:**

Compliance to anti-TB treatment is crucial in achieving cure and avoiding the emergence of drug resistance. Electronic health (eHealth) interventions are included in the strategy to end the global Tuberculosis (TB) epidemic by 2035. Evidences showed that mobile messaging systems could improve patient adherence to clinic appointment for diagnosis and treatment. This review aimed to assess the effect of mobile-phone messaging on anti-TB treatment success.

**Methods:**

All randomized controlled trial (RCT) and quasi-experimental studies done prior to August 26, 2019 were included in the review. Studies were retrieved from PubMed, EMBASE, Cochrane and ScienceDirect databases including, grey and non-indexed literatures from Google and Google scholar. Quality of studies were independently assessed using Cochrane Risk of Bias Assessment Tool. A qualitative synthesis and quantitative pooled estimation were used to measure the effect of phone messaging on TB treatment success rate. PRISMA flow diagrams were used to summarize article selection process.

**Results:**

A total of 1237 articles were identified, with 14 meeting the eligibility criteria for qualitative synthesis. Eight studies with a total of 5680 TB patients (2733 in intervention and 2947 in control groups) were included in meta-analysis. The pooled effect of mobile-phone messaging revealed a small increase in treatment success compared to standard of care (RR 1.04, 95% CI 1.02 to 1.06), with low heterogeneity (I^2^ = 7%, *p* < 0.0002). In the review, performance, detection and attrition biases were reported as major risk of biases.

**Conclusions:**

Mobile-phone messaging showed a modest effect in improving anti-TB treatment success; however, the quality of evidence was low. Further controlled studies are needed to increase the evidence-base on the role of mHealth interventions to improve TB care.

**Protocol registration number:**

CRD420170744339. http://www.crd.york.ac.uk/PROSPERO/display_record.php?ID=CRD42017074439

## Background

Globally, mobile cellular subscription coverage is growing exponentially [1]. The unprecedented spread and advancements in innovative application of mobile technologies is an opportunity to address health priorities [2]. Some mobile health applications have shown improvement in patients’ adherence to medications [3]. Evidences demonstrated that mobile-phone text messaging were effective in ART programs [4–7]. Electronic Health interventions has now brought attention in the strategy to end the global TB epidemic by 2035 [8]. In 2015, World Health Organization (WHO) established its Global Task Force on Digital Health for TB to advocate and support the development of digital health innovations in global efforts to improve TB care and prevention [9]. Electronic based patient education was components of a WHO conceptual framework for digital health in the TB response. This was intended to be accomplished using video (virtually) observed therapy [10], Short Message Service (SMS) and eLearning [11].

Tuberculosis is one of the critical public health threat which is the leading cause of mortality worldwide [12]. WHO estimated that globally one-third of the world population harbors latent TB infections [13]. An estimated 10 million TB cases in 2017 and the 30 High Burden Countries (HBCs) accounted about 90% of the global TB burden since 2015 [14].

Compliance to anti-TB treatment is crucial in achieving cure and avoiding the emergence of drug resistance (MDR- and XDR-TB).

Regular and complete medication intake gives individual TB patients the best chance of cure and also protects the community from the spread of TB [15]. One untreated infectious tuberculosis patient is likely to infect 10 to 15 persons annually [16]. The consequences of poor adherence to long-term therapies are poor health outcomes and increased health care costs interventions aimed at improving adherence would provide a significant positive return on investment through primary prevention and secondary prevention of adverse health outcomes [17–22].

Evidences showed that mobile messaging systems could improve patient adherence to clinic appointment for diagnosis and treatment [23]; TB prevention and promotion of Anti-TB treatment adherence [24, 25]. But evidences were limited on effect of phone messaging intervention specifically on Anti-TB treatment success. According to WHO, Anti-TB treatment success is defined as the sum of cured and treatment completed. The cure rate is defined as the percentage of patients who completed treatment and were culture negative during the last month of treatment and on at least one other occasion for non-MDR-TB and who had at least 5 consecutive results in the previous 12–15 months for MDR-TB. Whereas, completion rate is the percentage of MDR-TB and non-MDR-TB patients who completed treatment according to guidelines but did not meet the definition for cure or treatment failure due to a lack of bacteriological results [26]. According to the latest treatment outcome, the global treatment success rate was 82% in 2016 that showed a reduction from 86% in 2013 and 83% in 2015 [27].

The output of this review could be useful evidence for policy makers, researchers, care providers, community members and patients to consider in due plan and implementations. This systematic review was aimed:

- ❖ To appraise existing evidences of SMS to improve Anti-TB Treatment Success Rate (TSR)
- ❖ To assess pooled effect of SMS to improve Anti-TB TSR
- ❖ To assess the effect of frequency of messaging to improve TSR
- ❖ To identify type of SMS (reminder/educational messaging) more important for TSR.

## Methods

The protocol has been registered at PROSPERO with ID: CRD420170744339; http://www.crd.york.ac.uk/PROSPERO/display_record.php? ID = CRD42017074439.

### Criteria for considering studies for this review

#### Types of study

This study included RCT and Quasi-experimental studies comparing DOTS with and without mobile-phone reminder for patients on Anti-TB treatment. Studies written only in English were include in the review. Studies that measured treatment success including treatment completeness and cure were included in the in the qualitative synthesis. For meta-analysis, we included studies reported treatment success rate and studies reported data elements that enabled us to calculate treatment success rate.

#### Types of participants

TB patients aged ≥15 years were included in the review.

#### Types of interventions

Studies that used mobile-phone based messaging service for patients on DOTS as interventions group and those patients on DOTS without messaging was control groups.

#### Information source and search strategies

In this systematic review, databases including PubMed, EMBASE, Cochrane, and ScienceDirect were searched. In addition, grey literature and non-indexed articles were searched on Google and Google scholar. Searching strategies were based on Population, Intervention, Comparison, and Outcome (PICO) criteria and using Medical Subject Headings (MeSH), Boolean search operators, truncation symbols to robust the searching scheme. Searching was held for articles disseminated up to August 26, 2019. Please see the MeSH searching terms (Additional file 1).

### Study selection

Searched results were exported to Endnote X7 citation manager, duplicates were removed and studies were examined based on selection criteria. Abstracts of studies were examined for eligibility for meta-analysis and the full text of articles was searched when abstracts did not provide sufficient information to make a decision. Searching was independently carried out by two reviewers (KDG and ZAM) to reduce selection bias.

### Data collection process

In this review, data collection and analysis were based on the Cochrane Handbook of Systematic Reviews for Interventions [28].

### Risk of bias assessment

Quality of studies were independently assessed by two authors (KDG and ZAM) using Cochrane Risk of Bias Assessment Tool including: use of random sequence generation; concealment of allocation to conditions; blinding of participant and personnel; blinding of outcome assessors; completeness of outcome data and other; selective reporting and other biases. Each study was rated as low risk of bias when there was no concern regarding bias; as high risk of bias when there was concern regarding bias; or unclear risk of bias if the information was absent [28]. The level of agreement between the two reviewers were measured using Cohen’s Kappa level of agreement [29]. Third body was planned to decide if the two reviews couldn’t able to agree on a certain point of bias assessment.

### Data extraction and analysis

All shortlisted articles were independently reviewed by two reviewers and decided to include in the systematic review and then to the meta-analysis. Both authors (KDG and ZAM) independently extracted the selected measures of Anti-TB treatment success. Cure and treatment completeness were directly used for measuring TB treatment success. Treatment success was also indirectly estimated from unsuccessful treatment outcomes like, death, loss to follow-up, treatment failure and transfer-out. Cochrane Collaboration Review Manager Version 5.3 software was used for data extraction and analysis.

### Effect size determination

Anti-TB treatment success rate is measured by adding patients cured and those completed their treatment [26]. The pooled effects of intervention (mobile-phone messaging) was measured by using risk ratios (RR) and with 95% confidence intervals. The statistical heterogeneity between studies and its impact on meta-analysis was examined using the I^2^ statistic. Where, I^2^ (0% = no, 25% = low, 50% = moderate, 75% = high heterogeneity of effect sizes) [28]. A fixed and random effect models were used to estimate the RR (95% CI) based on the level of heterogeneity (I^2^) of the included studies [30, 31]. Sensitivity and subgroup analyses were used to identify the potential effect of heterogeneity of studies was evaluated using.

### Sensitivity analysis

Effect size analysis was run with and without the outlier to assess its effect on the overall findings. To assess whether individual studies had impact on summary effect, summary analysis was undertaken after removing each studies effect [28].

### Subgroup analysis

Four subgroups were pre-determined based on a priori chosen sub-variables aimed to understand their independent contributions in enhancing mobile-phone messaging to improve Anti-TB treatment success. These sub-variables were: purpose of messaging which classified as medication reminder and educational messaging; disease burden which classified as High Burden Countries (HBCs) and Non-HBCs; type of mobile-phone messaging used (Text, call, graphic reminder, etc.) and frequency of messaging (Daily, Weekly & Both Daily & Weekly).

### Assessment of publication bias

Publication bias was assessed using a funnel plot. When asymmetry was indicated by the funnel plot of the effect sizes by their standard error, the impact on the summary effect size was assessed. Egger’s test was used to see statistical test of publication bias. When an outlier was detected, the relevant study was re-examined.

### Rating quality of evidence

The Grading of Recommendations Assessment, Development, and Evaluation (GRADE) system was used to rate the quality of evidence of this review. Five factors, namely; limitations, inconsistency, indirectness, imprecision, and publication bias were used to rate the quality of evidence [32].

## Results

### Selection of studies

**Fig. 1:**
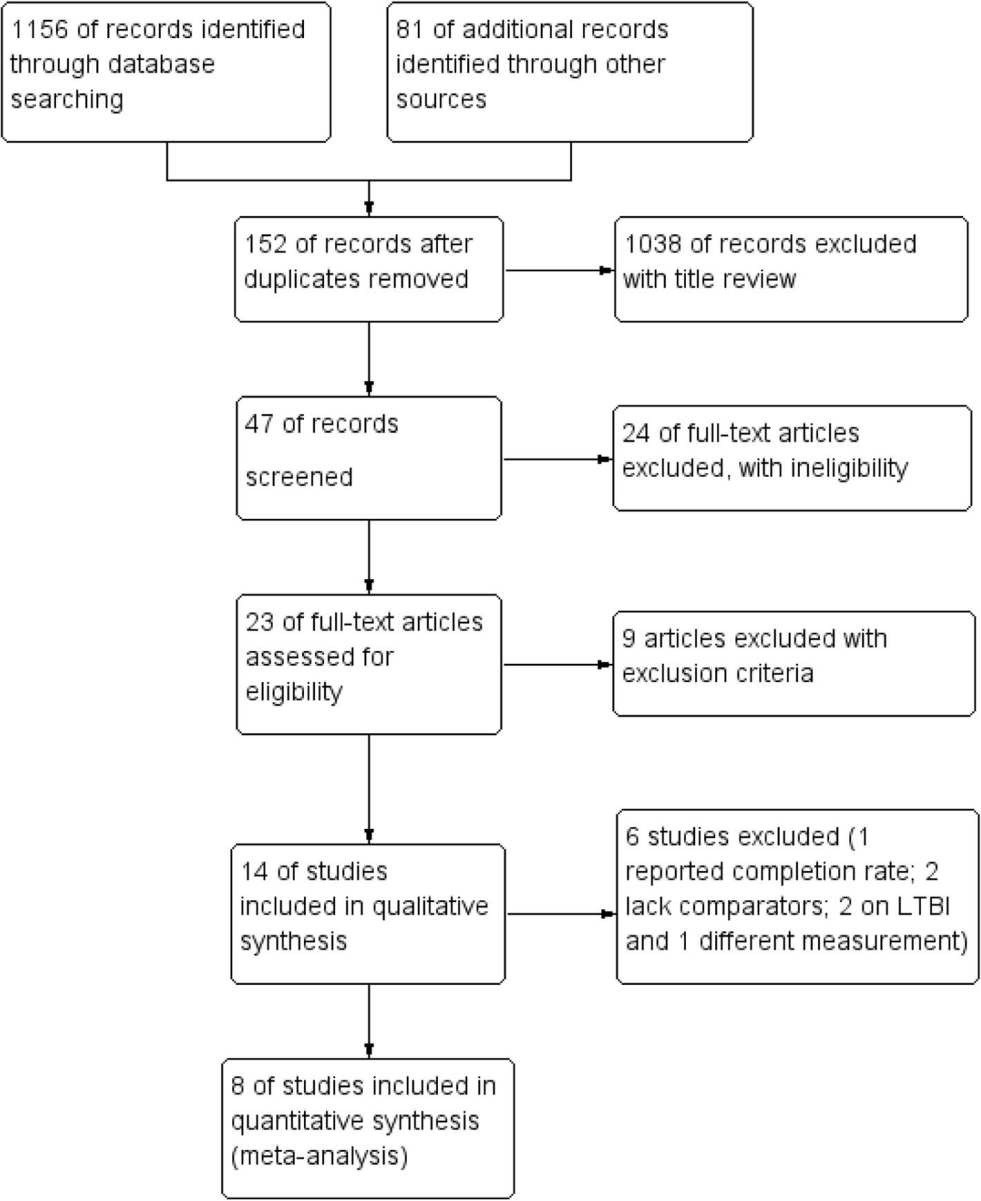
PRISMA flow diagram for inclusion of articles

Articles were retrieved from databases including PubMed (552), EMBASE (344), ScienceDirect (168), Cochrane (92) and grey literature from Google Scholar (78) and reference lists (3), as shown in the flow diagram for selection processes (Fig. 1).

From the total of 1237 articles retrieved, 152 articles excluded due to duplication; 1039 excluded with title review and 24 articles excluded due to ineligibility. Finally, 23 articles were fully read and 9 studies were excluded from the review with different reasons, as shown in Table 1. Some articles were excluded because of using video-based directly observed therapy [33–37] the intervention was different from phone text, audio, graphic and video messages. SMS intervention has an unrelated purpose [38] and study participants were not matching with this review [5, 39, 40].

**Table 1:** Characteristics of excluded studies

Study	Reasons for exclusion
Garfein, et’al, 2015 [33]	Different intervention (Video Based Directly Observed Therapy)
Holzschuh, et’al, 2017 [34]	Video Based Directly Observed Therapy
Buchman, et’al, 2017 [35]	Different intervention (Skype Observed Therapy)
Hoffman, et’al, 2010 [36]	Remote (video) mobile Direct Observation of Treatment
DeMaio, et’al, 2001 [37]	Focus on videophone technology
Lorent, et’al, 2014 [38]	SMS was used for issuing of test results not for treatment reminder
Bassett, et’al, 2016 [39]	Study subjects were HIV/TB co-infected patients
Chaiyachati, et’al, 2013 [5]	Intervention for Health care workers
Howard, et’al, 2016 [40]	Participants were HIV/TB co-infected

### Characteristics of selected studies

After exclusion of ineligible studies, fourteen studies were selected and reviewed for the qualitative synthesis of evidence. Among included studies, four in Africa, seven in Asia, one in South in one in North America, and one study enrolled patients from North America, Europe, Asia, and Africa. See details in Table 3 in the Appendix.

All studies implemented mobile phone messaging interventions on top of the standard TB treatment and care. The various types of phone messaging interventions were applied. Nine of fourteen studies used text-based phone messaging, two studies used only phone call and two studies applied a text and/or voice messages. One study implemented text and graphic messages for TB patients to adhere to their treatment. Among all studies, the messaging intervention was interactive (two-way) in seven studies, one-way in five and two studies didn’t report on the messaging model. The ultimate purpose of mobile messaging in 11 studies were to remind patients towards their anti-TB medication. In 3 studies the messaging intervention aimed to identify patients’ concerns, educate and motivate patients to engage on their own medication.

A study in Thailand [41] reported a significant effect of SMS messaging on TB treatment success rate. Whereas, other randomized control study in Argentina [42], in Pakistan [43, 44], in China [45] found no significant difference between the SMS or control groups for treatment success.

Two studies didn’t calculate significance test on the effect of SMS messaging on TSR of TB due to inappropriate or lack of comparator group [46, 47]. One study reported only about the level of patient adherence on TB treatment but not on treatment outcome [48]. The characteristics of each selected studies have explained in Table 3. Two randomized controlled studies in USA, Spain, Hong Kong and South Africa [49], and in Canada [52], reported that phone messaging did not significantly improve LTBI completion rates compared to standard care. Successful treatment of Latent Tuberculosis Infection (LTBI) also plays key role in eliminating TB [55]; however, difficult to ascertain treatment success in LTBI cases.

### Quantitative synthesis of evidence

Only eight studies [41–45, 50, 53, 54] were found to be fitted for the pooled estimation of the effect of mobile phone messaging on successful TB treatment outcome. Six studies were excluded with the following reasons. One study reported treatment completion and unable to calculate the treatment success rate from the reported data [51]. Two studies focused on LTBI treatment completion [49, 52] couldn’t be pooled to determining treatment success. One study used a different tool to measure the outcome variable [48] and studies excluded due to lack of comparison groups [46, 47].

#### Risk of bias assessment for the included studies

**Fig. 2:**
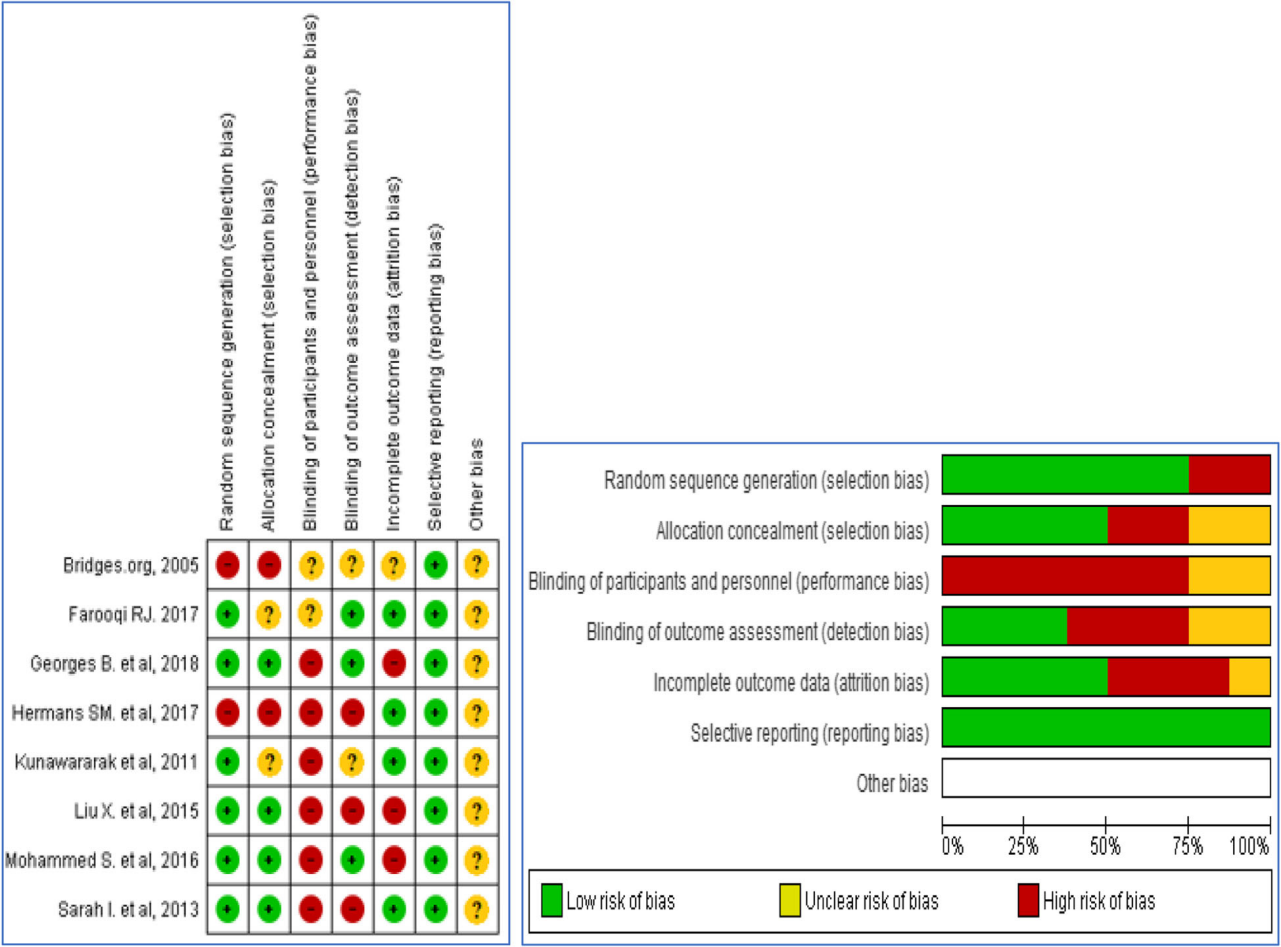
Risk of bias summary and graph: authors’ judgments for each included study

Among the selected studies for meta-analysis, performance bias was the major challenge in the studies, because of the nature of the intervention, participants could not be blinded. Blinding of outcome assessors was also major gap that could lead to detection bias. All studies were jugged free of selection and reporting bias. Two studies have attrition bias (Fig. 2). Based on Cohen’s Kappa level of agreement, the two reviewers (KDG and ZAM) have shown 83.3% agreement with k = 0.686, *p* < 0.0001 for the included papers.

#### Pooled estimation on the effect of phone messaging on TB treatment success

**Fig. 3:**
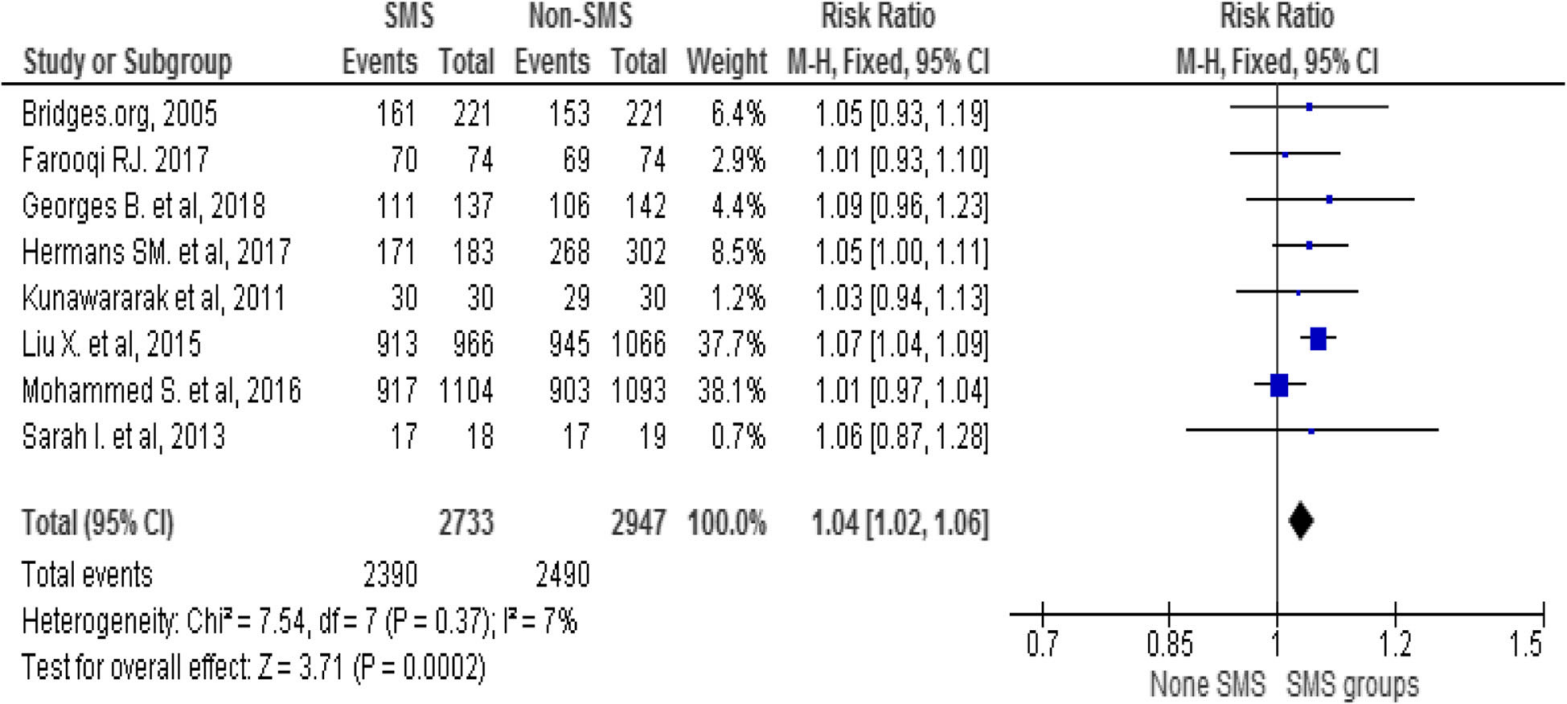
Forest Plot of the effect of mobile-phone messaging on Anti-TB treatment success

Overall, 5680 TB patients, 2733 in intervention and 2947 in control groups were involved in the pooled analysis. The overall Anti-TB treatment success was 2390/2733 (87.4%) in intervention and 2490/2947 (84.5%) in control groups with heterogeneity level (I^2^ = 7%, *p* < 0.0002). Fixed-effect model has shown that phone messaging group had higher treatment success rate compared to standard care (RR 1.04, 95% CI 1.02 to 1.06), see the Forest plot in Fig. 3.

**Fig. 4:**
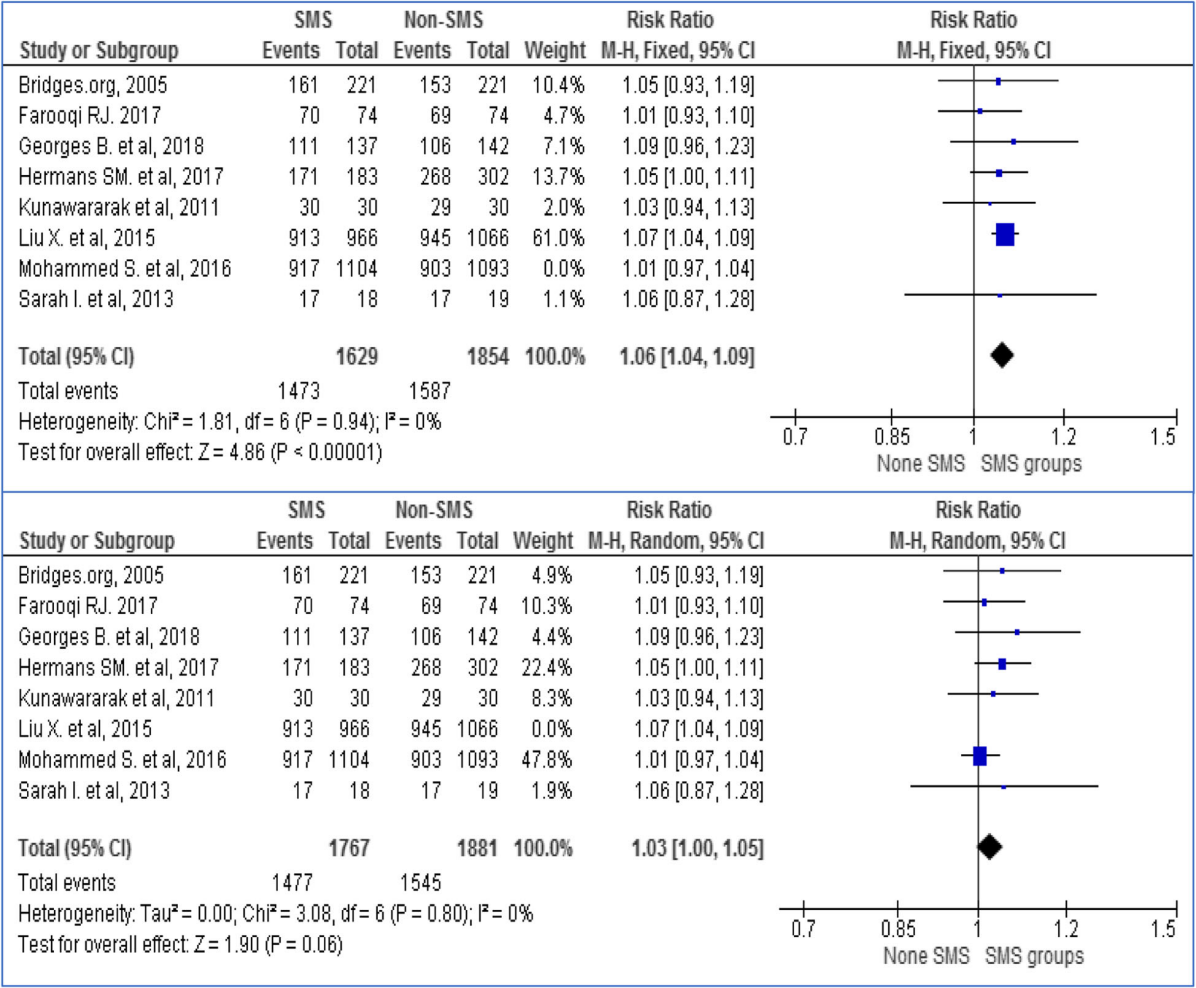
Sensitivity analysis excluding Mohammed et al. and Liu X. et al. studies to see its influence on effect size estimation

Sensitivity analysis was carried out to see the effect size of each model by excluding every one of the studies. All studies were taken out of the analysis one by one and the output indicated that no single study separately influenced the pooled effect of phone messaging on TB treatment success. The Forest plot for sensitivity analysis for the two big weighted studies (Mohammed et al. and Liu et al) presented on Fig. 4.

#### Sub-group analysis

**Fig. 5:**
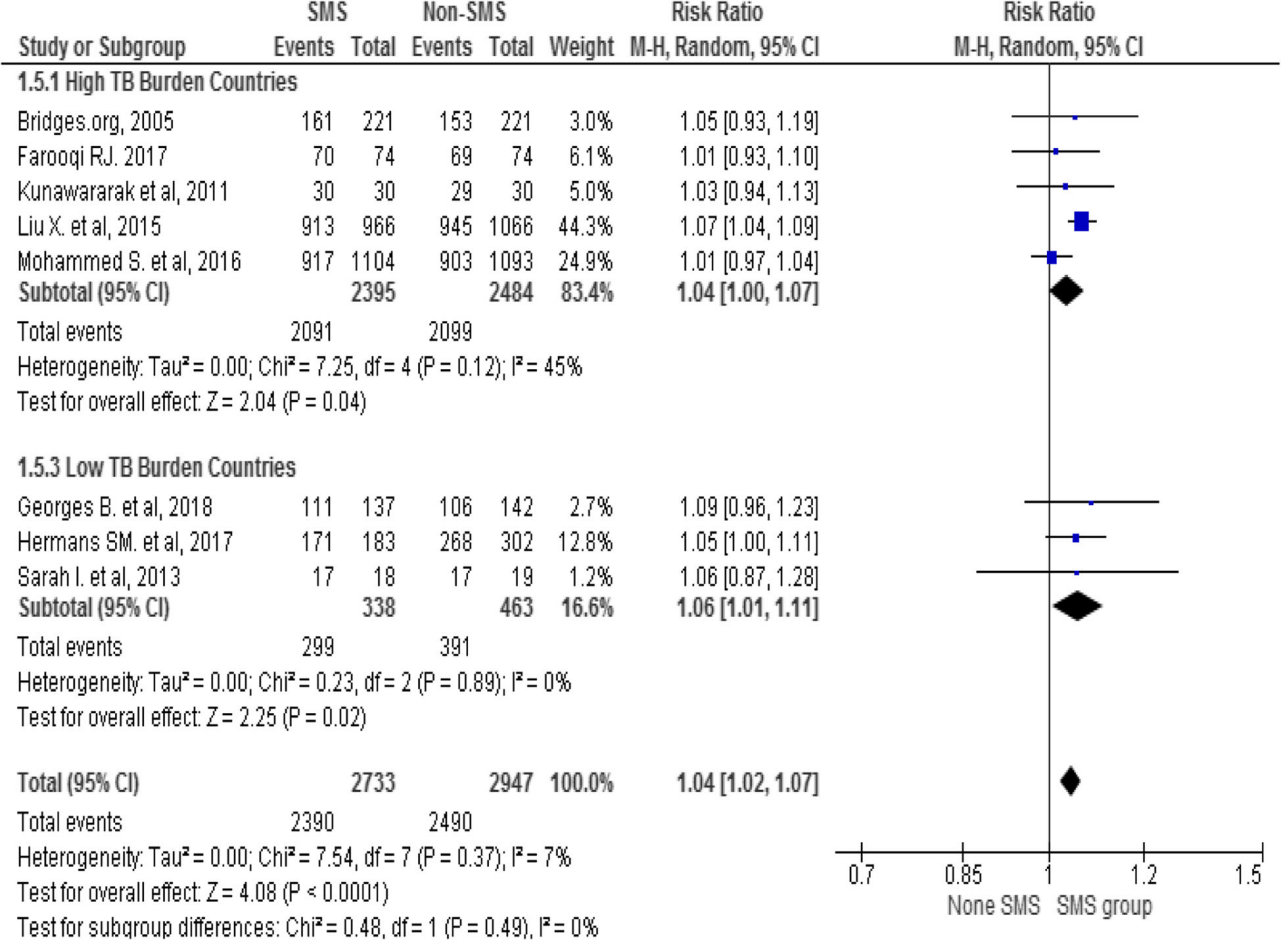
A sub-group analysis of effect of mobile-phone messaging on TB TSR by national TB burden

Sub-group analysis was carried out to identify the effect of interventions by the level of national TB burden (high Vs low) and model of phone messaging (one-way Vs two-way) applied by individual studies. The finding indicated that phone messaging has a modest effect on TSR in high TB burden (RR = 1.04; 95% CI 1.00–1.07) and in low TB burden countries (RR = 1.06; 95% CI 1.01–1.11) with moderate (I^2^ = 53%) and no (I^2^ = 0%) heterogeneity of studies in both groups respectively see Fig. 5.

**Fig. 6:**
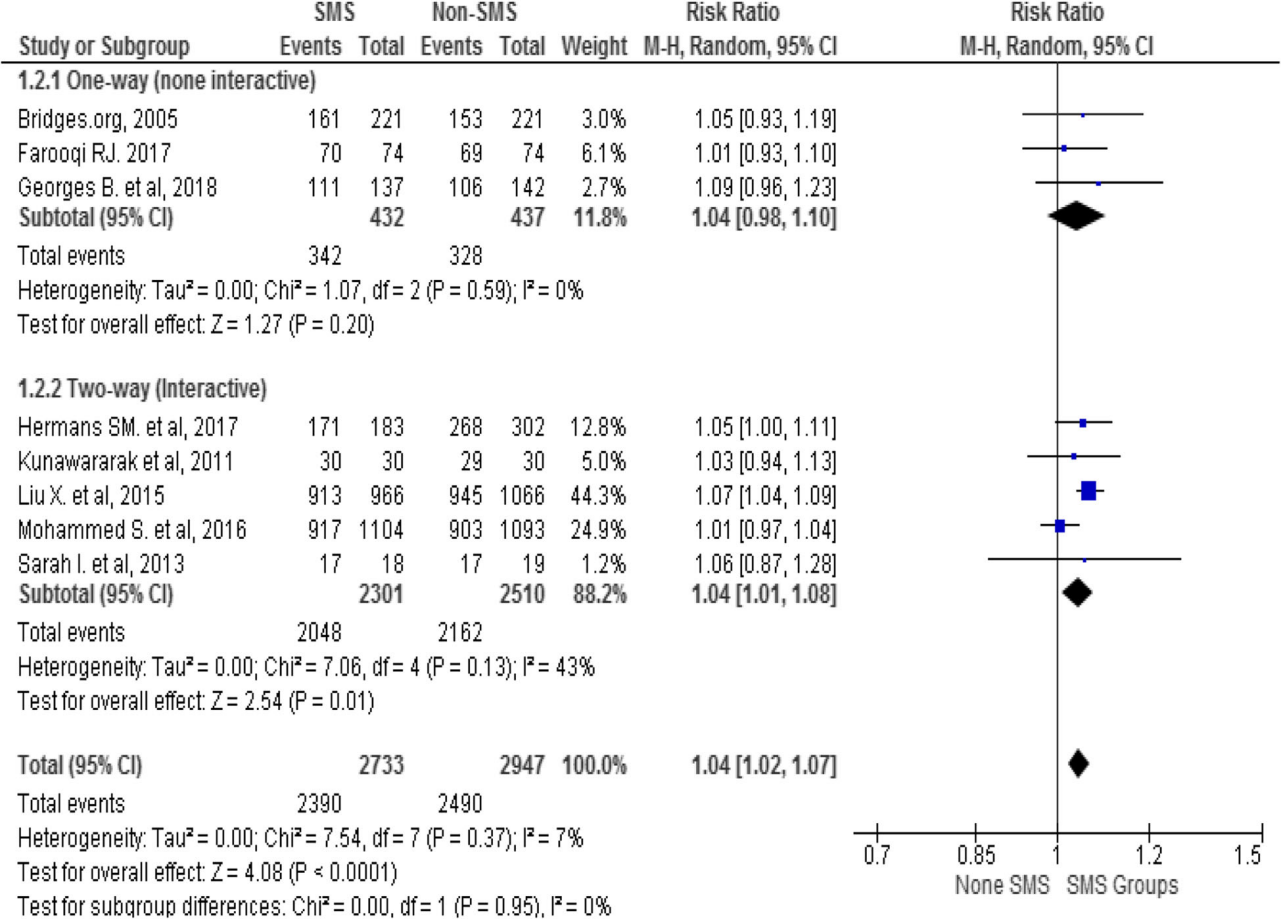
Effect of mobile-phone messaging on TB TSR sub-grouped by model of phone messaging

Similarly, a sub-group analysis by model of phone messaging (one-way Vs two-way) shown that interactive phone messaging has modest effect on TSR of TB (Fig. 6). Further sub-group analyses (purpose of the messaging, frequency of messaging, type of messaging and national income) were not conducted due to unable to get sufficient studies in the pre-determined sub-groups.

#### Publication bias

**Fig. 7:**
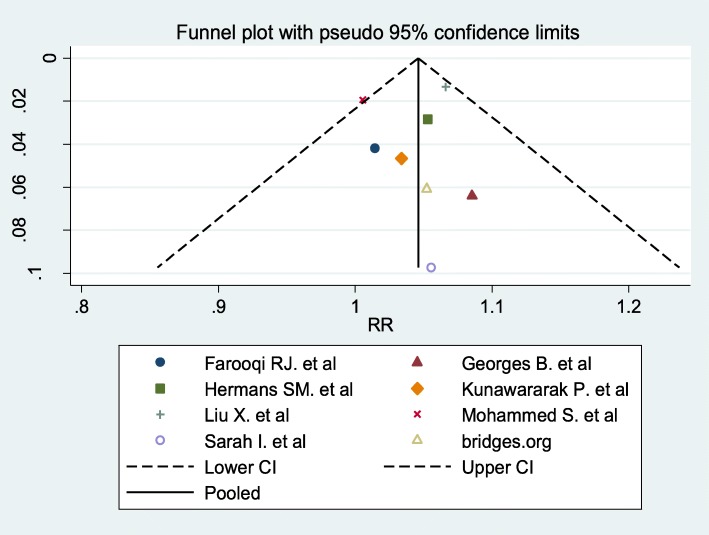
Funnel Plot of studies comparing phone messaging and standard of care on TSR of TB

A subjective visual inspection of funnel plot of the included studies pointed out symmetrical observation that shows less likely occurrence of publication bias. We used Egger’s test to objectively measured publication bias. The finding revealed that there was no evidence of publication bias (*P* = 0.753), (Fig. 7).

#### Rating quality of evidence

Based on GRADE quality of evidence assessment approach, the overall quality of evidence of this review was rated low, mainly due to limitations of performance, detection and selection biases for more details see Table 2.

**Table 2:** GRADE rating of the quality of evidence

Outcomes	№ of participants (studies) Follow up	Certainty of the evidence (GRADE)	Relative effect (95% CI)	Anticipated absolute effects^a^ (95% CI)	
TB treatment success assessed with: 6–9 months	5680 (8 Studies)	⊕ ⊕ ⊝⊝ LOW ^b, c^	RR 1.04 (1.02 to 1.06)	Assumed risk	
				Usual care group	Risk difference with phone Messaging
				901 per 1000	36 more per 1000 (18 more to 54 more)

^a^The risk in the intervention group (and its 95% confidence interval) is based on the assumed risk in the comparison group and the relative effect of the intervention (and its 95% CI). *CI* Confidence interval, *RR* Risk ratio

^b^High performance bias, detection and selection biases

^c^Some studies used self-assessment tools that subjectively measure treatment completeness

## Discussion

The substantial growth of mobile phones throughout the world has brought opportunities for integrating mobile phones as a health care intervention tools for TB patients [56]. Mobile Health is now inevitable to play big role to end the global TB epidemic [57]. In this review majority of the studies were obtained from Asia followed by Africa. About 64% (9/14) the studies implemented text-based phone messaging. Seven (50%) of the studies used interactive (two-way) approach.

In the current review, the pooled treatment success rate of TB was higher 87.4% in the intervention group than 84.5% in the control group. The finding conveyed slight growth from the latest global treatment success rate of 82% in 2016 [27]. The variation could be due to the recent studies that included the review. The meta-analysis has shown that phone messaging group had a modest increment in treatment success rate compared to standard care (RR 1.04, 95% CI 1.02 to 1.06) with low heterogeneity (I^2^ = 7%, *p* < 0.0002) between individual studies based on the cutoff value [28]. Similar systematic reviews showed that text-messaging interventions were effective for self-management of diabetes, weight loss, smoking cessation, physical activities and medication adherence for antiretroviral therapy [58, 59]. In the current review, the effect size was low as compared to evidence on ART. The variation could be mHealth has been well studied on ART. Whereas, evidence on the effect of mHealth on TB treatment remains limited [60–62]. The result implies that mobile-phone messaging has a promising effect in improving Anti-TB treatment success; however, further implementation science studies may require to endorse impact, usability and sustainability of mHealth interventions on supporting the global TB epidemic.

In this review, a sub-group analysis indicated that phone messaging has a modest effect on TB treatment success rate in both high and low TB burden countries. The impact could be from the growing penetration of mobile phone technology and mHealth initiatives globally including high disease burden and resource limited countries [56, 57]. The subgroup analysis has shown that two-way phone messaging interventions have a small effect on TB treatment success as compared with one-way messaging. Evidence also indicated that SMS interventions for ART that run two-way SMS communication were more acceptable than non-interactive reminders [59]. An interactive communication could provide an opportunity for patients to get their voices heard, and improve relationships and enhance engagement in their treatment.

This systematic review has shown that mobile-phone messaging could be encouraging approach to improve Anti-TB treatment success, however, paucity of high-quality evidence to direct policies. Detection, performance and attrition biases were observed in most of the studies included in this review.

The overall quality of evidence of this review was rated low by using GRADE approach. The main reasons were biases and low sample size in many of the studies. As a limitation, this systematic review and meta-analysis was reliant on inadequate number of studies. The review has merely included studies written in English.

## Conclusion

This systematic review shown paucity of high-quality evidences concerning effect of mobile-phone messaging on anti-TB treatment success. Mobile-phone messaging showed a modest effect in improving anti-TB treatment success, however the quality of evidence was low. Further controlled studies are needed to increase the evidence-base on the role of mHealth interventions to improve TB care. This systematic review will be updated as new evidence emerged.

### Implications for practice

Digital communication technologies including mHealth has promising impact on healthcare. The need to support further innovative mHealth initiatives from all stakeholders.

### Implications for future research

Current evidence is of low quality. This implies that further well designed and reported RCT studies are needed for a better quality of evidence on Anti-TB treatment outcome.

### Supplementary information

**Additional file 1.** The MeSH search terms used for systematic review and meta-analysis on effect of phone messaging on ant-Tb treatment outcome.

## Data Availability

All the data were presented within the manuscript.

## Appendix

**Table 3:** Characteristics studies included in the review

Study ID	Belknap, et al, 2017 [49]
Setting	USA, Spain, Hong Kong and South Africa
Methods	A randomized clinical trial
Participants	Adults (aged ≥18 years) LTBI cases
Sample size	Overall, 1002 LTBI case were participated
Interventions	A one-way weekly text-based treatment reminder
Outcomes	Treatment completion was 87% in DOTS, 74% in self-administration and 76 in self-administration plus SMS reminders groups.
Limitations	All participants were not randomly assigned to receive SMS reminders
Study ID	Barclay, 2009 [46]
Setting	South Africa
Methods	A single arm interventional study from July, 2006 to April, 2007
Participants	Tuberculosis patients at three clinics in the Cape Town
Sample size	155 patients
Interventions	Text messaging using SIMpill for 10 months
Outcomes	Drug adherence stabilized between 86 and 92% with a treatment success rate of 94% after patients used the SIMpill for 10 months.
Limitations	No comparator groups
Study ID	bridges.org, 2005 [50]
Setting	South Africa
Methods	A single arm trial with historical control
Participants	All adults with pulmonary and extra pulmonary TB were included in the study
Sample size	About 221 TB patients were followed in single arm design
Interventions	A one-way text-based daily phone reminder for anti-TB medication for six and eight months
Outcomes	Treatment success rate was 73% in trial and 69% in the latest statistics available for the City of Cape Town’s TB Control Program
Limitations	No inferential statistics in treatment success rates, due to limited sample size
Study ID	Fang X.H. et al, 2017 [51]
Setting	China
Methods	A Randomized controlled trial was conducted from December 1, 2014 to 31, 2015
Participants	All pulmonary TB patients from six districts
Sample size	Overall 350 (160 in intervention and 190 in control groups)
Interventions	A one-way text-based daily phone reminder for anti-TB medication for six months
Outcomes	The treatment completion rate in SMS group (96.25%) was significantly higher than that in the control group (86.84%), p-0.002
Limitations	Study included few predictor variables and generalizability restricted to one province
Study ID	Farooqi et’al, 2015 [44]
Setting	Pakistan
Methods	Randomized controlled trial was conducted from June 2014 to June 2015
Participants	Patients enrolled for anti-TB drugs were distributed in intervention and control groups
Sample size	148 TB patients
Interventions	A one-way text and graphic reminders sent daily to intervention group for two months
Outcomes	Primary outcome was default, defined as not taking medicine for two consecutive months. TB treatment success rate was 96.9% in intervention group and 94.26% in controls, p-0.983
Limitations	Didn’t assess background knowledge of participants
Study ID	Johnston, et al, 2018 [52]
Setting	Canada
Methods	A parallel, randomized controlled trial
Participants	Adults initiating LTBI therapy between June 2012 and September 2015
Sample size	Overall, the study enrolled 358 participants (170 in intervention and 188 in control arms)
Interventions	An interactive (two-way) text and phone call message service for LTBI adherence.
Outcomes	Treatment completion was 79% in intervention and 82% in control groups with RR 0.97; *p* = 0.550
Limitations	Outcome influenced by intensive monitoring schedule of the standard care
Study ID	Georges B. et al, 2018 [53]
Setting	Cameroon
Methods	A randomized controlled trial conducted between February 2013 and April 2014
Participants	Adults (> 18 years) and newly diagnosed PTB patients
Sample size	Two hundred seventy-nine participants; 137 in intervention 142 in control groups
Interventions	A one-way daily text-based reminder and motivational messages for six months
Outcomes	At five months, treatment success was 81% in intervention and 75% in control groups with OR = 1.45; *p* = 0.203.
Limitations	High attrition of participants
Study ID	Hermans SM. et al, 2017 [54]
Setting	Uganda
Methods	Quasi-experimental study design held between November 2010 and October 2011
Participants	Adult, literate, HIV/TB patients access with mobile phone
Sample size	Overall 485 (183 in intervention and 302 in control groups) followed up.
Interventions	An interactive (two way) text-based medication and appointment reminder, and educational messages using a total of 8 SMSs per 2 weeks for two months
Outcomes	After 8 weeks intervention, successful completion of treatment was 93% in intervention and 89% in control groups, p-0.43.
Limitations	Use of pre-intervention control group prone to temporal changes could influence outcomes
Study ID	Kumboyono, 2017 [48]
Setting	Indonesia
Methods	A post-test-only controlled-group design
Participants	Adult TB patients enrolled on treatment
Sample size	45 TB patients enrolled on treatment
Interventions	A text-based phone messaging to motivate patients
Outcomes	There was no difference in treatment compliance between the SMS and control groups with a p-0.059 of Fisher’s Exact test.
Limitations	Limited sample size
Study ID	Kunawararak et’al, 2011 [41]
Setting	Thailand
Methods	A two arm RCT between April 2008 and December 2009
Participants	New sputum smear positive pulmonary TB patients (both non-MDR-TB and MDR-TB) Patients aged > 15 years
Sample size	98 (60 Non-MDR and 38 MDR) TB patients
Interventions	An interactive daily phone call reminder for six and eighteen months
Outcomes	Treatment success Rate (TSR) was significantly higher in intervention group (100%) than control (96.7%) in non-MDR-TB, (p-0.047).
Limitations	Limited sample size
Study ID	Liu et’al, 2012 [45]
Setting	China
Methods	A pragmatic cluster-randomized trial in 36 districts. From 1 June 2011 to 7 March 2012, 4292 TB patients were enrolled across the clusters.
Participants	New pulmonary TB patients, starting on standard 6-month short-course chemotherapy
Sample size	4292 TB patients
Interventions	An interactive daily text messages to reminder medications for six months
Outcomes	TB treatment success was 96.1% in SMS groups, 91.4% in control groups, with p-0.084
Limitations	Over-estimation of poor adherence
Study ID	Mohammed et’al, 2013 [43]
Setting	Pakistan
Methods	A two-arm, randomized controlled trial in Karachi, Pakistan. Individual participants were randomized to either SMS or the control group.
Participants	Newly-diagnosed patients with smear or bacteriologically positive pulmonary tuberculosis who were on treatment for less than two weeks; ≥15 years; reported having access to a mobile phone; and intended to live in Karachi throughout treatment were eligible. The study enrolled 2207 participants, with 1110 randomized to SMS and 1097 to the control group.
Sample size	2207 TB patients
Interventions	An interactive daily text pill reminder for six months and participants respond with SMS or missed calls after taking medication. Up to 3 SMSs sent for non-respondents a day.
Outcomes	There was no significant difference between the SMS or control groups for treatment success (719 or 83% vs. 903 or 83%, respectively, *p* = 0.782).
Limitations	Lack of an objective tool to measure adherence
Study ID	Narasimhan et’al, 2013 [47]
Setting	India
Methods	Single arm interventional study
Participants	TB patients seeking care from the DOTS centers
Sample size	104 patients recruited, 100 patients were followed until end of treatment
Interventions	Text and/or voice call reminder enabled treatment adherence support system
Outcomes	A voice call reminder system could improve patients adherence to TB drugs
Limitations	The effect size of the intervention was not determined
Study ID	Sarah I. et al, 2013 [42]
Setting	Argentina
Methods	A randomized 1:1 allocation
Participants	Patients newly diagnosed with TB who were ≥ 18 years, and had mobile-phone access
Sample size	38 TB patients (18 in intervention and 19 in control)
Interventions	An interactive bi-weekly text-based educational messages to patients to adhere to medication for the first 2 months of treatment
Outcomes	Treatment success was 17/18 in intervention arm and 17/19 in control arm.
Limitations	Baseline knowledge not addressed; use self-reporting that may bias the outcome

## Abbreviations

GRADE: Grading of Recommendations Assessment, Development and Evaluation
HBCs: High Burden Countries
MeSH: Medical Subject Headings
PICO: Population, Intervention, Comparison and Outcome
PRISMA: Preferred Reporting Items for Systematic Reviews and Meta-Analyses
RCT: Randomized Controlled Trial
SMS: Short Message Service
TB: Tuberculosis
