# Supplementary material for: Does phone messaging improves tuberculosis treatment success? A systematic review and meta-analysis
Source: BMC Infect Dis. 2020 Jan 14;20:42. doi: 10.1186/s12879-020-4765-x (PMC6961375; doi:10.1186/s12879-020-4765-x)
Supplement: Supplementary file 1 — Additional file 1. The MeSH search terms used for systematic review and meta-analysis on effect of phone messaging on ant-Tb treatment outcome. [file 12879_2020_4765_MOESM1_ESM.docx]

**Additional file 2**

The MeSH search terms used for systematic review and meta-analysis on effect of phone messaging on ant-Tb treatment outcome.

*((((((((Tuberculosis[MeSH Terms]) OR Tuberculosis[All Fields]) OR TB[All Fields]))*

*AND*

*(((((((Treatment[MeSH] OR Treatment[ALL Fields] OR Treatment outcome[MeSH Terms]) OR Treatment outcome[All Fields]) OR Treatment Failure[MeSH Terms]) OR Treatment Failure[All Fields]) OR Treatment completeness[All Fields]) OR Treatment success[All Fields]OR DOTS[All Fields])))))))*

*AND*

*((((((((Text Messaging[MeSH Terms]) OR Text Messag*[All Fields]) OR Telemedicine[MeSH Terms]) OR mhealth[All Fields]) OR Reminder system[MeSH Terms]) OR Reminder system*[All Fields]) OR cellphone communicat*[All Fields] OR SMS[All Fields] OR Mobile Health[All Fields] OR Messag*[All Fields] OR Remind*[All Fields])))*
